# Cross-reactive antibodies elicited to conserved epitopes on SARS-CoV-2 spike protein after infection and vaccination

**DOI:** 10.1038/s41598-022-10230-y

**Published:** 2022-04-20

**Authors:** Eric S. Geanes, Cas LeMaster, Elizabeth R. Fraley, Santosh Khanal, Rebecca McLennan, Elin Grundberg, Rangaraj Selvarangan, Todd Bradley

**Affiliations:** 1 Genomic Medicine Center, Children’s Mercy Research Institute, Kansas City, MO USA; 2 Department of Pediatrics, University of Missouri- Kansas City, Kansas City, MO USA; 3 Department of Pediatrics, University of Kansas Medical Center, Kansas City, MO USA; 4 Department of Pathology and Laboratory Medicine, University of Kansas Medical Center, Kansas City, KS USA; 5 Department of Pathology and Laboratory Medicine, Children’s Mercy, Kansas City, MO USA

**Keywords:** Infectious diseases, Viral infection, Medical research, Translational research, Immunology, Adaptive immunity, Humoral immunity, Antibodies

## Abstract

SARS-CoV-2 is a novel betacoronavirus that caused coronavirus disease 2019 and has resulted in millions of deaths worldwide. Novel coronavirus infections in humans have steadily become more common. Understanding antibody responses to SARS-CoV-2, and identifying conserved, cross-reactive epitopes among coronavirus strains could inform the design of vaccines and therapeutics with broad application. Here, we determined that individuals with previous SARS-CoV-2 infection or vaccinated with the Pfizer-BioNTech BNT162b2 vaccine produced antibody responses that cross-reacted with related betacoronaviruses. Moreover, we designed a peptide-conjugate vaccine with a conserved SARS-CoV-2 S2 spike epitope, immunized mice and determined cross-reactive antibody binding to SARS-CoV-2 and other related coronaviruses. This conserved spike epitope also shared sequence homology to proteins in commensal gut microbiota and could prime immune responses in humans. Thus, SARS-CoV-2 conserved epitopes elicit cross-reactive immune responses to both related coronaviruses and host bacteria that could serve as future targets for broad coronavirus therapeutics and vaccines.

## Introduction

Coronaviruses are large, enveloped, positive-stranded RNA viruses with the defining feature of corona-like spike proteins across the cell surface^1^. The Severe Acute Respiratory Syndrome Coronavirus 2 (SARS-CoV-2) is a novel coronavirus shown to be the cause of Coronavirus Disease 2019 (COVID-19)^2,3^. A large focus of SARS-CoV-2 research has been on the spike protein, which binds to the host cell receptor and mediates entry into host cells^4^. The spike protein consists of two functional subunits: the S1 subunit containing the receptor binding domain (RBD) for binding to the Angiotensin Converting Enzyme 2 (ACE2) receptor, and the S2 subunit responsible for fusion of viral and host cell membranes^4,5^. Neutralizing antibodies in immune responses to SARS-CoV-2 frequently bind to these subunits of the coronavirus and could prevent infection^6–13^. Importantly, the spike protein and its functions have been evolutionarily conserved between other coronaviruses^9,14–17^.

Prior to the SARS-CoV-2 outbreak in 2019, there had been outbreaks of coronaviruses such as Severe Acute Respiratory Syndrome Coronavirus (SARS-CoV-1) that infected individuals in 2003 and Middle East Respiratory Syndrome coronavirus (MERS-CoV) in 2012^18–22^. SARS-CoV-2 is a member of the betacoronavirus family and shares genetic similarities with prior coronaviruses that have caused outbreaks in humans as well as other emergent betacoronaviruses that have been identified in nonhuman hosts such as bats and pangolins, which are common sources of animal to human transmissions (Bat Coronavirus RaTG13 and Pangolin Coronavirus identifier QIQ54048.1)^23,24^. In addition to coronaviruses with pandemic potential that could cause severe disease, there are seasonal coronaviruses that readily circulate and cause mild disease each year. The first two identified were seasonal alphacoronavirus Human CoV 229E and betacoronavirus Human CoV OC43^25,26^. Betacoronavirus Human CoV HKU1 and alphacoronavirus Human CoV NL63 have since become part of the common seasonal coronavirus strains regularly circulating with Human CoV 229E and Human CoV OC43^27–30^. Due to the increased frequency of novel coronaviruses that could infect humans, there is an urgent need to develop more broad coronavirus therapeutics and vaccines.

There are currently three SARS-CoV-2 vaccines in the United States (U.S.) that are either approved by or authorized for emergency use by the U.S. Food and Drug Administration (FDA); Pfizer-BioNTech's BNT162b2, Moderna's mRNA-1273, and Johnson & Johnson's Janssen JNJ-78436735^31–33^. These vaccines utilize messenger ribonucleic acid (mRNA) or adenovirus platforms to deliver genetic information for the expression of the SARS-CoV-2 spike protein for presentation to the host immune system^34^. All three vaccines have been shown to elicit robust humoral and cellular immune responses and have demonstrated efficacy at preventing severe COVID-19^10,31–33,35–39^.

Multiple studies have identified that individuals without previous SARS-CoV-2 infection had varying levels of pre-existing antibodies that could cross-react with SARS-CoV-2 and may influence SARS-CoV-2 immunity and disease severity^40–44^. Furthermore, other groups have identified regions on the SARS-CoV-2 spike that are highly conserved and could serve as targets to generate more broad cross-reactive immunity to many types of coronaviruses^45,46^. These naturally occurring or elicited poly-reactive antibodies could recognize phylogenetically closely related viruses and could potentially provide immunity against emerging viral variants or related strains. With an increase in novel coronaviruses infecting humans in recent history, pan-coronavirus vaccines targeting a multitude of related viruses would be an effective, possibly necessary, tool against future viral threats. Previous studies with other viral targets have exemplified that immunization to a virus or antigen could provide a broad antibody response and cross-reactivity against similarly structured antigens regardless of viral origin^47,48^. It will be critical to identify and characterize epitopes on SARS-CoV-2 that are the source of cross-reactive immune responses.

In humans, there is considerable variability in the immune responses to both infections and vaccinations within the population. Several genetic and environmental factors have been identified that could help explain the mechanisms of this variation. One potential source of cross-reactive immunity, apart from viruses with similar protein sequence or structure, could be exposure to commensal bacteria that contain proteins with sequence homology to infecting viruses. Components of the host gut microbiome have been shown to prime or alter the immune system response to vaccination prior to exposure to antigens^49,50^. Recently, epitopes within the SARS-CoV-2 receptor binding domain and S2 subunits of the spike protein have been shown to share epitopes with commensal bacteria^51,52^. These shared epitopes may have primed immune responses to individuals naïve to the viral components. However, the origins of the SARS-CoV-2 cross-reactive antibodies and their potential impacts on vaccines and infection have not been fully elucidated.

In this study, we determined cross-reactive antibody responses elicited to related alpha- and betacoronaviruses after SARS-CoV-2 infection or COVID-19 BNT162b2 vaccination. Moreover, we identified immunodominant peptide epitopes within the SARS-CoV-2 spike protein with high sequence conservation amongst betacoronaviruses that could serve as platforms for future vaccine designs. Additionally, we identified that antibody responses to SARS-CoV-2 spike S2 subunit epitopes could cross-react with commensal gut bacteria proteins that may prime SARS-CoV-2 antibody responses^51,52^.

## Results

### Comparative analysis of closely related coronavirus spike proteins

**Figure 1:**
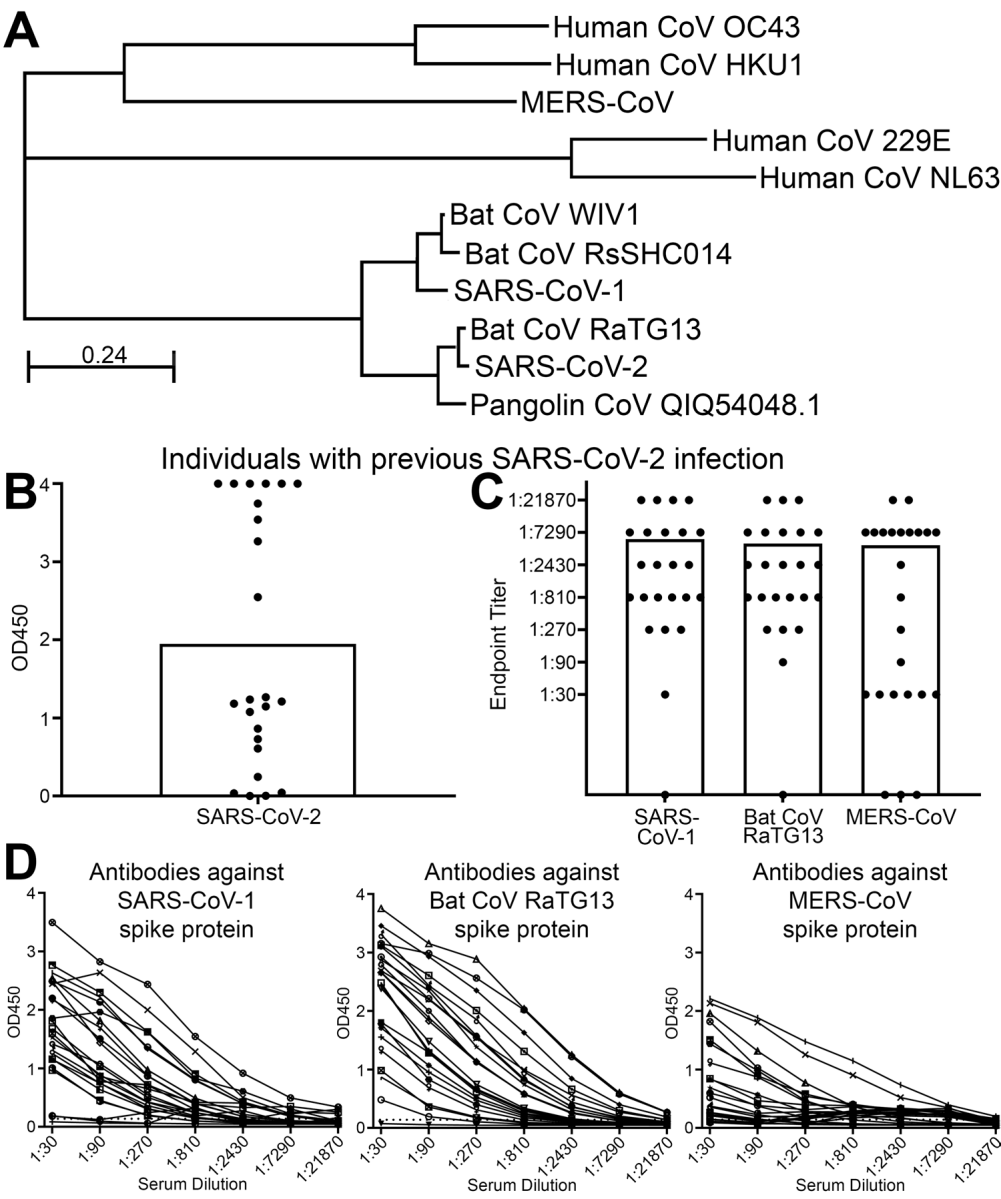
SARS-CoV-2 infection elicited cross-reactive antibodies towards other related viruses. (**A**) Phylogram of viral spike protein sequences of different alpha and betacoronaviruses. (**B**) Bar graph of OD450 absorbance values obtained by ELISA for determining SARS-CoV-2 IgG antibody responses using serum from convalescent individuals with recent SARS-CoV-2 infection. Each dot represents a distinct individual, n = 24. (**C**, **D**) Bar (**C**) and line graphs (**D**) of antibody responses obtained by ELISA at different serum dilutions to SARS-CoV-1, Bat CoV RaTG13 and MERS-CoV spike proteins using serum from individuals with recent SARS-CoV-2 infection. Endpoint titers are designated by the most dilute plasma concentration detected above the minimum threshold of the background OD450 multiplied by 3. Each dot or line represents a distinct individual, n = 24. Bars are representative of the mean of all samples.

We performed a Multiple Sequence Alignment (MSA) to determine the amino acid sequence similarities between the viral spike protein sequences of closely related coronaviruses to SARS-CoV-2 and generated a phylogenetic tree from the underlying alignment. Specifically, we compared the coronavirus spike protein sequences of SARS-CoV-1 and SARS-CoV-2 with four SARS-like coronaviruses that infect animals and are not yet zoonotic, as well as four human seasonal coronavirus spike protein sequences and MERS-CoV (Fig. 1A & Supplemental Fig. 1A). Newly emergent Bat CoV RaTG13 and Pangolin CoV QIQ54048.1 were closely related to SARS-CoV-2 while Bat CoV WIV1 and Bat CoV RsSHC014 were more closely related to SARS-CoV-1 (Fig. 1A). MERS-CoV was more distantly related to the SARS coronaviruses we compared (Fig. 1A). It is important to note that although MERS-CoV, SARS-CoV-1 and SARS-CoV-2 all belong to the betacoronavirus genus, the phylogenetic origin of MERS-CoV is classified as clade II while SARS-CoV-1 and SARS-CoV-2 are clade I, clusters IIb and IIa respectively^53,54^. With regards to the four seasonal human coronaviruses that generally cause moderate or mild respiratory symptoms, Human CoV 229E and Human CoV NL63 clustered together (both alphacoronaviruses) and Human CoV OC43 clustered with Human CoV HKU1 (both betacoronaviruses)^55,56^ (Fig. 1A). Thus, the spike proteins of SARS-CoV-1, SARS-CoV-2, and several emergent coronaviruses have high sequence similarity within the spike protein that is critical for host cell attachment and infectivity.

Next, we collected plasma from 24 individuals of diverse age, sex, and race/ethnicity who had laboratory confirmed SARS-CoV-2 infection 12–42 days prior to collection (Supplementary Table 1). Using an enzyme-linked immunosorbent assay (ELISA) that measured immunoglobulin (Ig) G antibody responses to the SARS-CoV-2 spike protein, we found that 20 of 24 individuals (83%) had detectable antibodies to SARS-CoV-2 (Fig. 1B). We then investigated whether the previously infected SARS-CoV-2 individuals (seropositive) had cross-reactive antibody responses to the related coronaviruses using ELISA to the spike proteins for SARS-CoV-1, the emergent Bat CoV RaTG13, and the more distantly related MERS-CoV. We found that 23 of 24 samples (96%) demonstrated detectable antibody response to SARS-CoV-1 and Bat CoV RaTG13 (Fig. 1C, D). Surprisingly, 21 of 24 samples (88%) also demonstrated an antibody response to the more distantly related MERS-CoV spike protein (Fig. 1C, D). These results suggested that infection with SARS-CoV-2 elicited antibody responses in recovered individuals that cross-reacted with other coronaviruses, even those belonging to different betacoronavirus clades.

### The Pfizer-BioNTech BNT162b2 vaccine elicited cross-reactive antibodies to related coronaviruses

**Figure 2:**
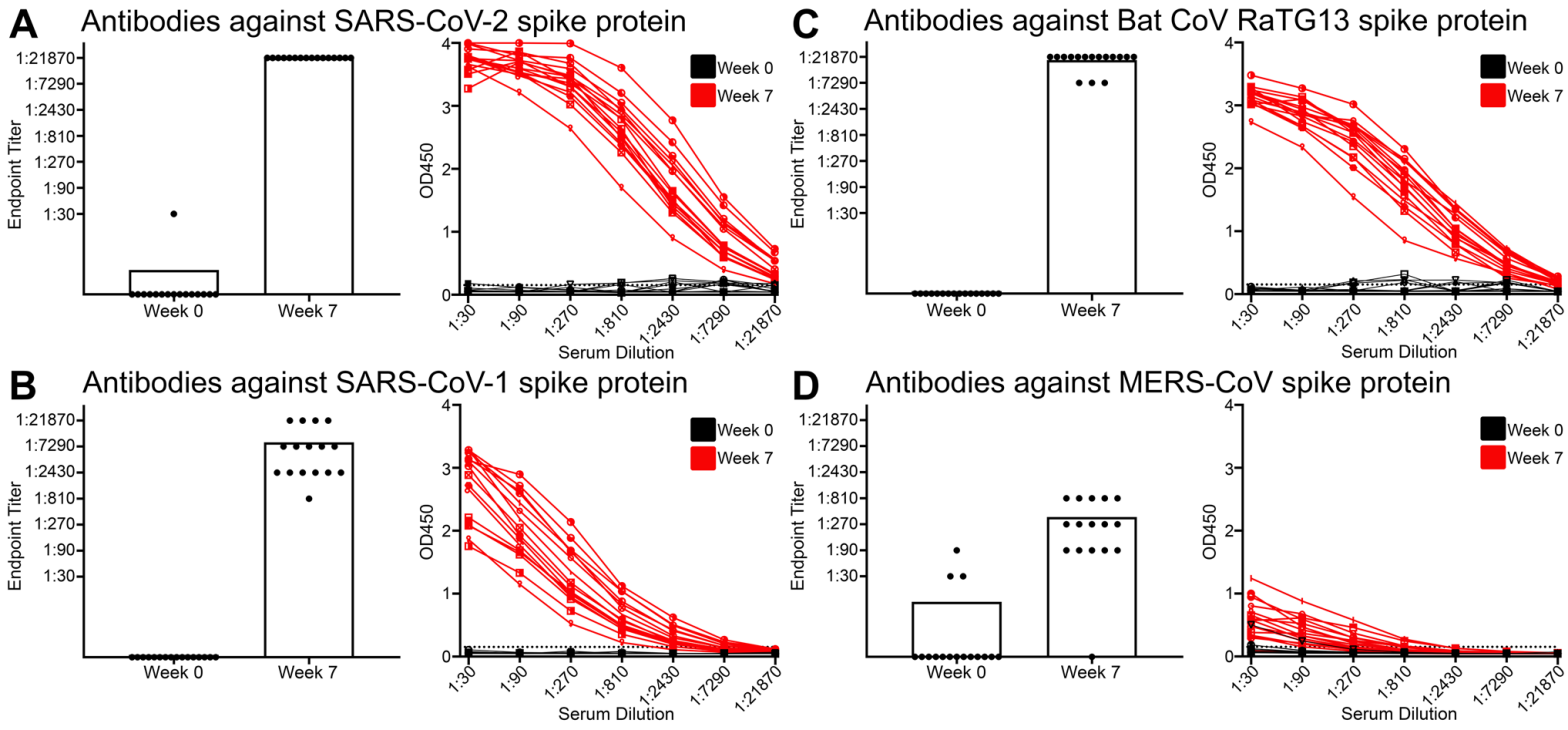
Vaccination targeting SARS-CoV-2 also elevated antibody responses to other related viruses. (**A**) Bar and line graph of antibody responses obtained by ELISA at different plasma dilutions to SARS-CoV-2 spike protein, before the Pfizer-BioNTech BNT162b2 vaccine was administered (Week 0) and after both doses (Week 7), using plasma from patients with no history of SARS-CoV-2 infection, n = 16 individuals. (**B**) Bar and line graph of antibody responses obtained by ELISA at different plasma dilutions to SARS-CoV-1 spike protein, before the Pfizer-BioNTech BNT162b2 vaccine was administered (Week 0) and after both doses (Week 7), using plasma from patients with no history of SARS-CoV-2 infection, n = 16 individuals. (**C**) Bar and line graph of antibody responses obtained by ELISA at different plasma dilutions to Bat CoV RaTG13 spike protein, before the Pfizer-BioNTech BNT162b2 vaccine was administered (Week 0) and after both doses (Week 7), using plasma from patients with no history of SARS-CoV-2 infection, n = 16 individuals. (**D**) Bar and line graph of antibody responses obtained by ELISA at different plasma dilutions to MERS-CoV spike protein, before the Pfizer-BioNTech BNT162b2 vaccine was administered (Week 0) and after both doses (Week 7), using plasma from patients with no history of SARS-CoV-2 infection, n = 16 individuals. Endpoint titers are designated by the most dilute plasma concentration detected above the minimum threshold of the background OD450 multiplied by 3. Dots are representative of individual samples. Bars are representative of the mean of all samples.

We next investigated whether vaccination with SARS-CoV-2 spike could elicit cross-reactive antibodies against different coronaviruses, similar to what was observed after natural infection. We collected plasma from individuals with no history of SARS-CoV-2 infection at two time points: 1) baseline before vaccine was administered (Week 0), and 2) four weeks after the two-dose immunization regimen with the Pfizer BNT162b2 mRNA COVID-19 vaccine was completed (Week 7) (Supplementary Table 2). We found that most individuals did not have detectable antibody levels, as measured by ELISA for SARS-CoV-2, SARS-CoV-1, Bat CoV RaTG13, or MERS-CoV spike proteins at week 0 (Fig. 2A–D). We did detect low levels of reactivity at week 0 in one individual against SARS-CoV-2 and three individuals against MERS-CoV, but only at the lowest plasma dilution (Fig. 2A, D). After both immunizations with the COVID-19 vaccine at Week 7, all individuals (16 of 16) had high levels of antibodies against SARS-CoV-2 spike protein (Fig. 2A). Moreover, all individuals also had detectable levels of cross-reactive antibodies against SARS-CoV-1 spike protein and to the emergent Bat CoV RaTG13 spike protein (Fig. 2B, C). Albeit detected at lower levels than SARS-CoV-1 and Bat CoV RaTG13, 15 of the 16 individuals had increased antibodies against the more distantly related MERS-CoV spike protein after vaccination with SARS-CoV-2 mRNA vaccine (Fig. 2D). These results suggested that 2 doses of the Pfizer-BioNTech BNT162b2 vaccine against SARS-CoV-2 was also effective at eliciting cross-reactive antibodies against other related coronaviruses.

### Pre-existing antibody levels targeting seasonal coronaviruses were largely not significantly affected by the Pfizer-BioNTech BNT162b2 vaccine

**Figure 3:**
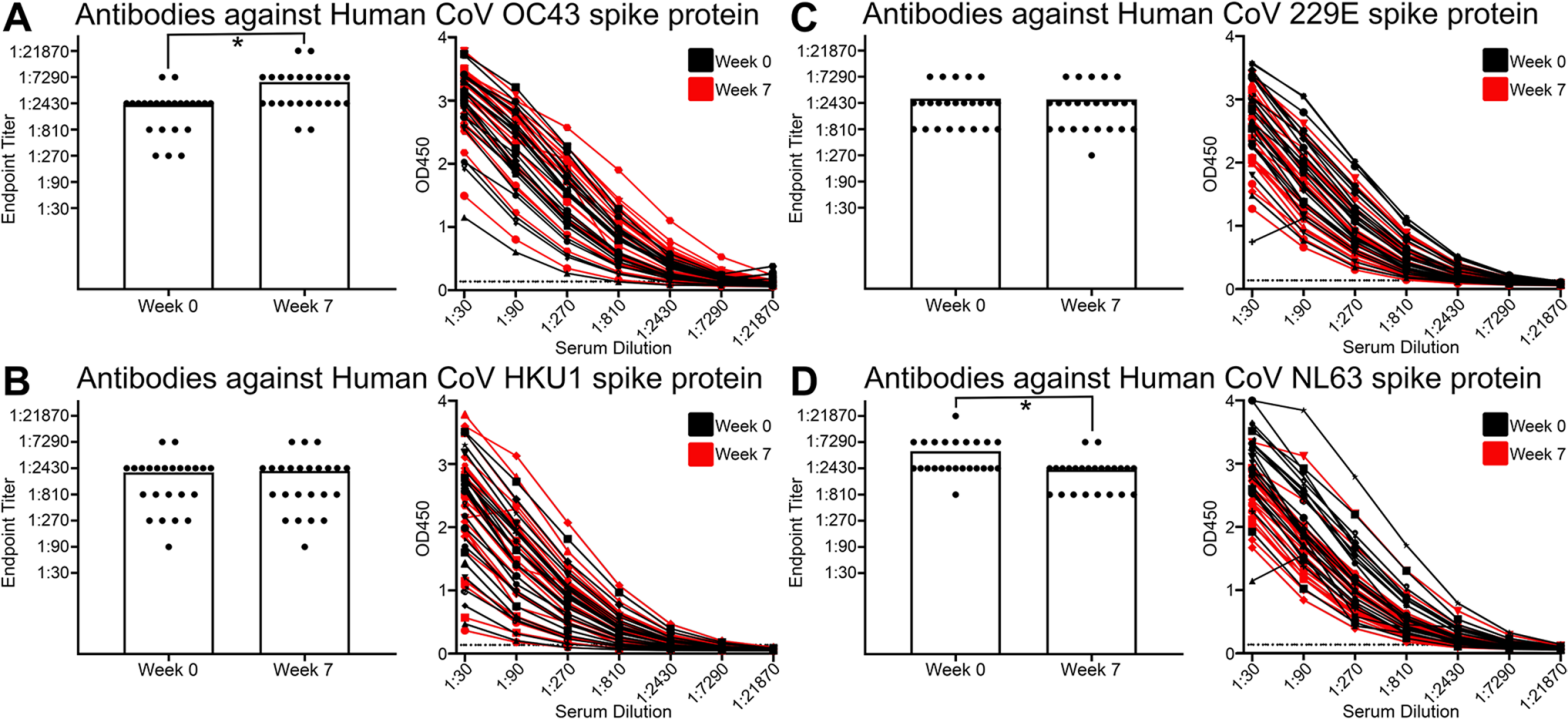
Vaccination targeting SARS-CoV-2 did not significantly affect antibody responses against most seasonal coronaviruses. (**A**) Bar and line graph of antibody responses obtained by ELISA at different plasma dilutions to Human CoV OC43 spike protein, before the Pfizer-BioNTech BNT162b2 vaccine was administered (Week 0) and after both doses (Week 7), using plasma from patients with no history of SARS-CoV-2 infection, n = 24 individuals, *p* ≤ 0.0001. (**B**) Bar and line graph of antibody responses obtained by ELISA at different plasma dilutions to Human CoV HKU1 spike protein, before the Pfizer-BioNTech BNT162b2 vaccine was administered (Week 0) and after both doses (Week 7), using plasma from patients with no history of SARS-CoV-2 infection, n = 24 individuals, *p* ≥ 0.99. (**C**) Bar and line graph of antibody responses obtained by ELISA at different plasma dilutions to Human CoV 229E spike protein, before the Pfizer-BioNTech BNT162b2 vaccine was administered (Week 0) and after both doses (Week 7), using plasma from patients with no history of SARS-CoV-2 infection, n = 24 individuals, *p* = 0.75. (**D**) Bar and line graph of antibody responses obtained by ELISA at different plasma dilutions to Human CoV NL63 spike protein, before the Pfizer-BioNTech BNT162b2 vaccine was administered (Week 0) and after both doses (Week 7), using plasma from patients with no history of SARS-CoV-2 infection, n = 24 individuals, *p* ≤ 0.0001. Endpoint titers are designated by the most dilute plasma concentration detected above the minimum threshold of the background OD450 multiplied by 3. Dots are representative of individual samples. Bars are representative of the mean of all samples. Statistical analysis was performed using Wilcoxon matched-pairs signed rank test with two-tailed P values reported.

Seasonal coronaviruses are more divergent in viral sequence to SARS-CoV-2 and are reasonably common, first infecting humans in childhood, and contribute to up to 30% of illnesses that are categorized as the common cold^57^. There are currently four major seasonal coronaviruses that infect humans, Human CoV 229E, Human CoV OC43, Human CoV HKU1 and Human CoV NL63. The amino acid sequence homology of spike proteins of the endemic coronaviruses to SARS-CoV-2 are less than 30% (Supplemental Fig. 1A). We measured antibody levels against the spike proteins from all four seasonal coronaviruses in the individuals with no history of SARS-CoV-2 infection at the two COVID-19 mRNA vaccine time points (week 0 and week 7 described above) from our hospital workers based in the United States. We found that all individuals (16 of 16) had detectable antibodies by ELISA to the spike proteins from all four seasonal coronaviruses prior to immunization at baseline (week 0), reflecting the high prevalence of pre-existing immunity to these four seasonal coronaviruses (Fig. 3A–D). After immunization with the COVID-19 mRNA vaccine (week 7), there was a modest but statistically significant increase in antibody magnitude in individuals against the Human CoV OC43 spike protein (Paired Wilcoxon *p* < 0.0001; Fig. 3A). However, there was no significant increase from baseline in antibody levels to the other three seasonal coronavirus spike proteins after SARS-CoV-2 mRNA vaccination (Fig. 3B–D). We did observe that Human CoV NL63 had a statistically significant decrease in antibody response (Paired Wilcoxon *p* < 0.0001; Fig. 3D), but the mechanism of this decrease is unclear. These data demonstrated that individuals had high levels of preexisting antibody levels against the four seasonal coronaviruses prior to COVID-19 vaccination and that the Pfizer-BioNTech BNT162b2 vaccine did not significantly boost the antibody responses to these coronavirus spike proteins, apart from the modest increase observed for Human CoV OC43.

### Identification of immunodominant regions with high sequence conservation on the SARS-CoV-2 spike protein

**Figure 4:**
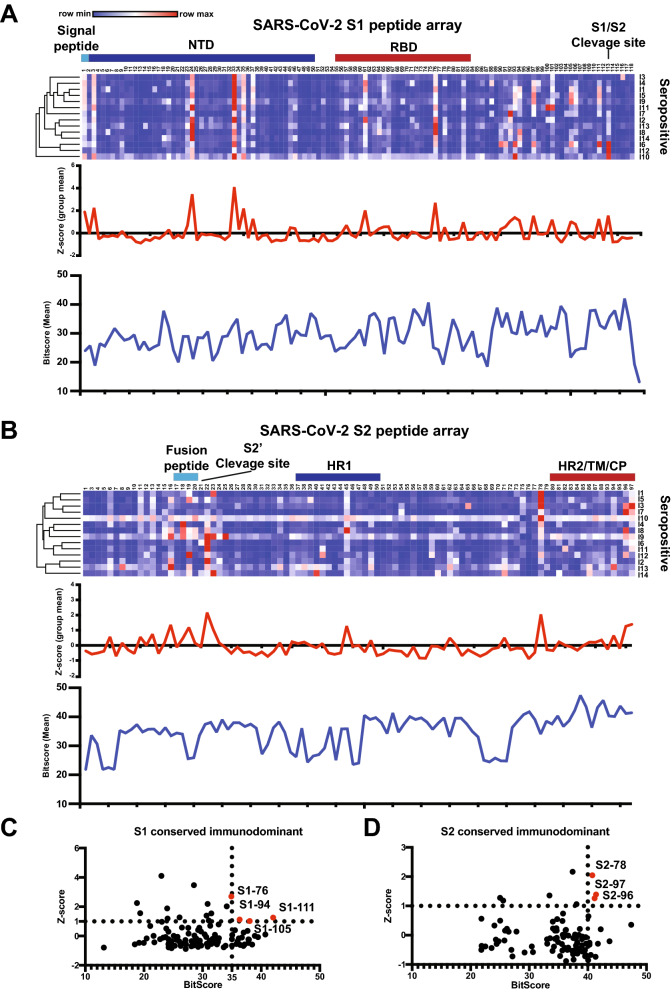
SARS-CoV-2 peptide array identified conserved immunodominant regions. (**A**, **B**) S1 (**A**) and S2 (**B**) subunits; 12-mer overlapping peptides). Seropositive (top) represents 14 individuals previously infected with SARS-CoV-2. Each peptide was printed in triplicate, and the Log2 of the mean fluorescent intensity (F635) for each peptide was graphed. The row color corresponds to the minimum and maximum intensity for all peptides for each individual. Known regions of the spike protein are annotated above the heatmaps. Line graphs display the mean group Z-score and mean Bitscore for each peptide. (**C**, **D**) Scatter plot of mean group Z-score versus mean Bitscore to identify putative immunodominant peptides for S1 (**C**) and S2 (**D**) subunits. NTD, N-terminal domain; RBD, receptor-binding domain; HR1, heptad repeat 1; HR2; heptad repeat 2; TM, transmembrane domain; CP, cytoplasmic domain.

Since we observed the elicitation of cross-reactive antibody responses against related coronaviruses after exposure to the SARS-CoV-2 spike protein, we next determined the precise antibody epitopes targeted after SARS-CoV-2 infection using a peptide microarray. Specifically, we performed a SARS-CoV-2 peptide array that included overlapping peptides across the S1 and S2 subunits of the SARS-CoV-2 spike protein using convalescent blood samples from 14 individuals previously infected with SARS-CoV-2 (Fig. 4A, B). We calculated z-scores across the group of individuals for each specific peptide and identified peptide regions in both the spike S1 and S2 subunits that were immunodominant among the 14 individuals. We considered a z-score greater than one as “immunodominant” as the levels would be one standard deviation higher than binding to the other peptides on the spike protein. These immunodominant regions were present in the RBD of the spike protein that is critical for recognizing the host cell receptor ACE2, but also outside of the RBD, in the N-terminal domain (NTD) of the S1, near the fusion peptide and the C-terminal of the S2 subunit (Fig. 4A, B). In order to measure sequence conservation among related coronaviruses for each peptide, we generated a Bitscore for each peptide from a BLAST sequence alignment using SARS-CoV-2, SARS-CoV-1, Bat CoV RaTG13, and MERS-CoV spike protein amino acid sequences (Fig. 4A, B). To identify immunodominant peptides that also had high sequence conservation among related coronaviruses, z-scores and Bitscores were compared in both the S1 and S2 regions of the SARS-CoV-2 spike protein (Fig. 4C, D). We identified four peptides in the S1 region (S1-76, S1-94, S1-105, S1-111) and three peptides in the S2 region (S2-78, S2-97, S2-96) that were all immunodominant (z-score ≥ 1) and had high sequence conservation with Bitscores one standard deviation above the mean (Fig. 4C, D). These results demonstrated that there are regions within the S1 and S2 subunit that are conserved at the amino acid level between multiple coronaviruses and are also frequently targeted by antibodies in individuals after infection with SARS-CoV-2.

### Immunization with conserved SARS-CoV-2 peptide epitope in mice elicited cross-reactive antibodies to multiple coronaviruses

**Figure 5:**
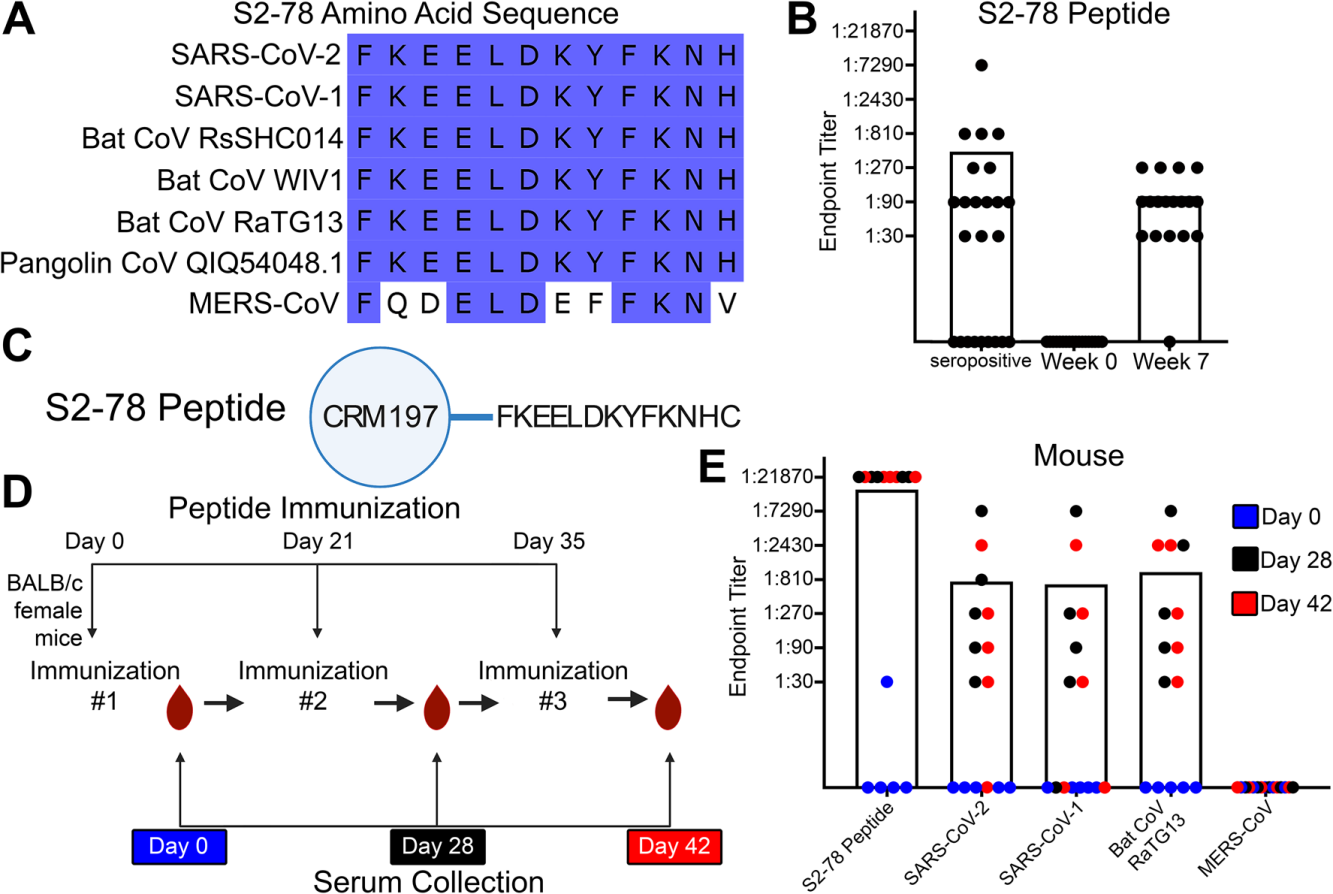
An immunodominant region of the S2 subunit elicited antibody responses against SARS-CoV-2 and other related viruses. (**A**) Amino acid sequence alignments of the S2-78 peptide across SARS-CoV-2, closely related SARS-CoV-1, three emergent bat coronaviruses (Bat CoV RsSHC014, Bat CoV WIV1, and Bat CoV RaTG13), an emergent pangolin coronavirus with the identifier QIQ54048.1 and MERS-CoV. Blue shading indicates amino acid conservation. (**B**) Bar graph of antibody responses (endpoint titers) obtained by ELISA at different plasma dilutions to S2-78 peptide in distinct vaccinated human seronegative week 0 and week 7 plasma (n = 16) and human seropositive serum (n = 24). (**C**) Schematic representation of the S2-78 peptide with the addition of the conjugated diphtheria toxin carrier protein CRM197 for immunization studies. (**D**) Timeline of the mouse experiments. (**E**) Bar graph of antibody responses (endpoint titers) obtained by ELISA at different serum dilutions of mouse serum against the S2-78 peptide, SARS-CoV-2, SARS-C0V-1, Bat CoV RaTG13 and MERS-CoV (n = 5 mice per each time point). Endpoint titers are designated by the most dilute serum concentration detected above the minimum threshold of the background OD450 multiplied by 3. Dots are representative of individual samples. Bars are representative of the mean of all samples.

One of the SARS-CoV-2 spike peptides that was both immunodominant after infection and displayed high sequence conservation was not in the RBD but instead was located at the C-terminal of the S2 (S2-78) (Fig. 4D). This region has been shown to have the potential for neutralization by antibodies after infection and antibodies targeting this region correlated with disease outcomes after SARS-CoV-2 infection^58–60^. Multiple sequence alignment of the S2-78 peptide sequence from SARS-CoV-2 with the closely related SARS-CoV-1, the three emergent bat coronaviruses (Bat CoV RsSHC014, Bat CoV WIV1, and Bat CoV RaTG13), and the emergent pangolin coronavirus with the identifier QIQ54048.1 revealed 100% amino acid sequence identity (Fig. 5A). The more distantly related MERS-CoV did not have identical conservation in the S2-78 peptide and differed in amino acids at 5 of the 12 positions (Fig. 5A). Similarly, there was little homology of S2-78 amino acid sequences to the seasonal human coronaviruses 229E, NL63 and HKU1 (Supplemental Fig. 1B). Surprisingly, there was 75% amino acid identity of S2-78 to the Human CoV OC43 seasonal coronavirus, despite the full spike protein of Human CoV OC43 having less than 30% amino acid sequence identity (Supplemental Fig. 1A and B).

Using ELISA, we found that 15 of the 24 seropositive individuals (63%), had high levels of antibody binding to the S2-78 peptide (Fig. 5B). None of the seronegative individuals that were vaccinated at week 0 demonstrated any antibody binding to the S2-78 peptide at baseline (Fig. 5B). However, after COVID-19 vaccination (week 7) all but a single individual (96%) had high levels of antibody binding to the S2-78 peptide (Fig. 5B). This confirmed the frequent targeting of this epitope after SARS-CoV-2 infection or vaccination.

We next sought to determine if immunization with the S2-78 peptide could elicit a cross-reactive antibody response to related coronaviruses. Since peptide antigens are often poorly immunogenic alone, we synthesized the S2-78 peptide and conjugated it with a diphtheria toxin carrier protein CRM197 to boost immunogenicity as a peptide-conjugate vaccine (Fig. 5C). We immunized five BALB/c mice with the S2-78 conjugate vaccine three times on days 0, 21 and 35 (Fig. 5D). We collected blood samples at baseline and seven days after each immunization at days 0, 28, and 42 (Fig. 5D). After the second (day 28) or third (day 42) immunizations we detected antibody binding to the S2-78 peptide for all five animals (Fig. 5E). Most mice also had detectable antibodies against the full SARS-CoV-2 spike protein (day 28, 5 of 5; day 42, 4 of 5) as well as the SARS-CoV-1 (day 28, 4 of 5; day 42, 3 of 5) and Bat CoV RaTG13 (day 28, 5 of 5; day 42, 5 of 5) spike proteins (Fig. 5E). We did not detect any antibodies in the mice targeting the more divergent MERS-CoV spike protein after immunization with the S2-78 peptide conjugate vaccine. We tested a single concentration of serum from each of the mice (1:40) in a SARS-CoV-2 and SARS-CoV-1 pseudovirus neutralization assay and did not detect a significant reduction in infection or neutralization (Supplemental Fig. 2). This may indicate that higher levels are required to determine neutralization or the other conserved epitopes with stronger neutralization activity could be selected for future vaccine designs. Thus, immunization with a peptide conjugate vaccine in mice using a peptide that is conserved elicited cross-reactive antibodies against multiple coronaviruses.

### Cross-reactivity with commensal gut bacteria may prime antibody responses to the S2 subunit of SARS-CoV-2

**Figure 6:**
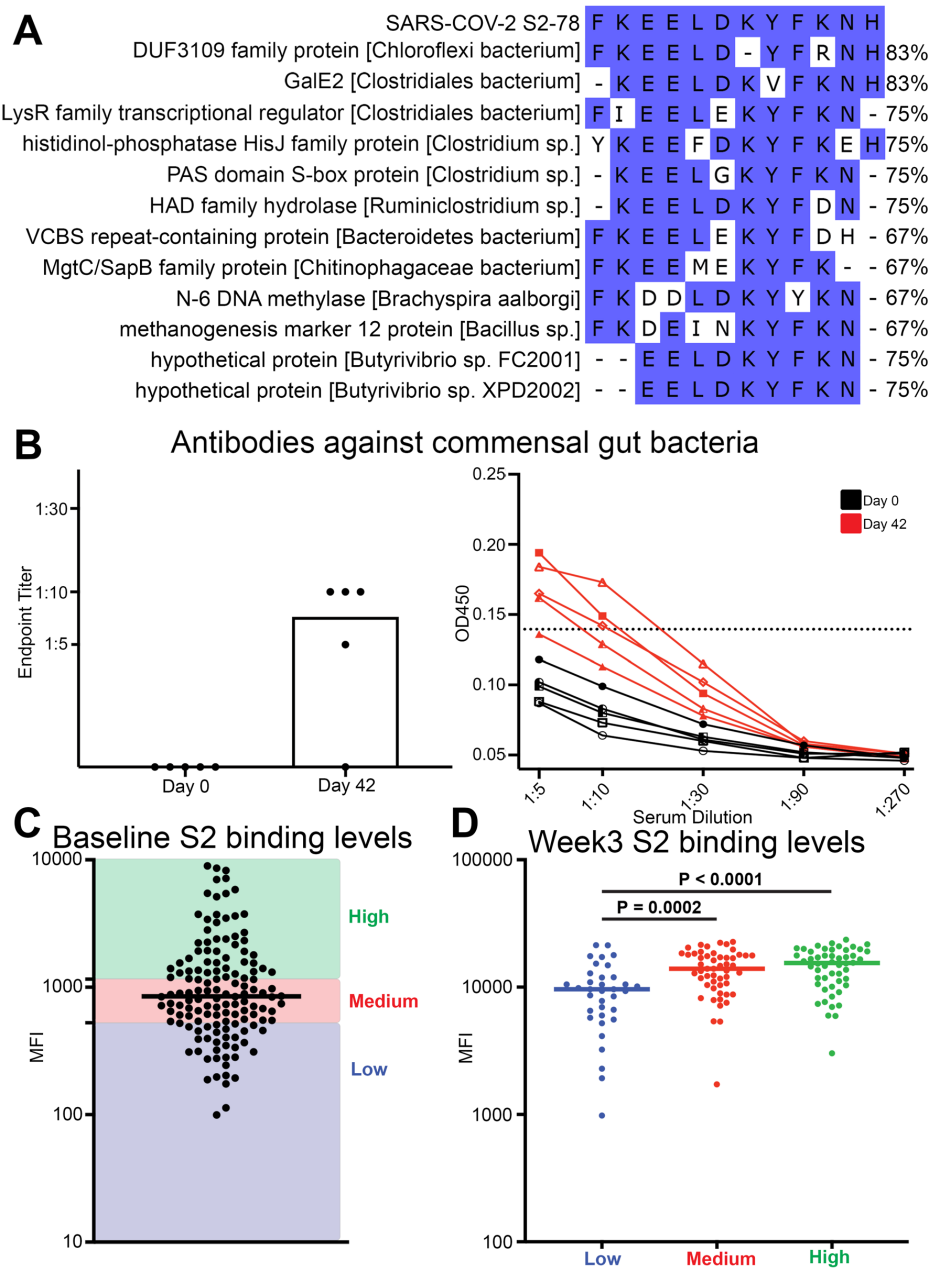
Immunodominant S2-78 peptide was cross-reactive with commensal gut bacteria. (**A**) Amino acid sequence alignments of the S2-78 peptide across 12 example bacterial sequences identified using sequence alignment of the bacteria proteome (Fig. 5A). Blue shading indicates amino acid conservation, dashes indicate gaps in sequence. Percentage of sequence homology shown on the right of the alignments. (**B**) Bar and line graph of antibody responses obtained by ELISA at different serum dilutions of mouse serum immunized with the S2-78 peptide against the commensal gut bacteria cell lysate (n = 5 mice per each time point). Endpoint titers are designated by the most dilute plasma concentration detected above the minimum threshold of the background OD450 multiplied by 3. Dots are representative of individual samples. Bars are representative of the mean of all samples. (**C**, **D**) Multiplex bead-based antibody binding assay that measured the IgG antibody response to SARS-CoV-2 spike subunit 2 (S2) in distinct healthy individuals (**C**) at baseline (n = 140) prior to receiving COVID-19 vaccine or (**D**) at week 3 after receiving the first COVID-19 vaccine immunization. Individual values of Median Fluorescent Intensity (MFI) are calculated; background subtraction has been used to remove nonspecific signal. Line indicates group median. Individuals at baseline with the top 75% MFI were grouped as high (green) and individuals with the lowest 25% MFI were grouped as low (blue) and individuals with 25–75% MFI were grouped as medium (red). P values were determined with a Wilcoxon–Mann–Whitney test.

A recent report showed that a region in the SARS-CoV-2 S2 subunit that contained the S2-78 peptide sequence elicited pre-existing antibodies that cross-reacted with commensal gut bacteria^51^. Using the SARS-CoV-2 S2-78 sequence as reference, we performed a sequence alignment against the entire bacterial proteome to identify sequence homology between the viral and commensal bacterial proteins that could be targeted by the humoral immune response. We identified numerous bacterial proteins with similar sequences that existed across a broad range of bacteria, including proteins from many genera of bacteria that exist within the human microbiome. Specifically, the top 10 bacterial proteins had sequence homology with SARS-CoV-2 S2-78 between 66.6 and 83.3%, which is more conserved than the 58.3% homology of distantly related MERS-CoV (Fig. 6A).

Serum from the mice immunized with the S2-78 peptide conjugate vaccine at baseline (day 0) and after three immunizations (day 42) was used to determine antibody cross-reactivity to commensal gut bacteria that was collected from human feces. We found that 4 of the 5 mice had increased antibody reactivity compared to baseline against the human fecal protein lysate after immunization at day 42 at either 1:5 or 1:10 serum dilutions (Fig. 6B). This data suggested that proteins contained in commensal gut bacteria shared protein sequence homology with the coronavirus S2-78 peptide and could provide preemptive exposure to cross-reactive epitopes without exposure to the coronavirus spike protein.

Interestingly, we observed heterogeneity in the magnitude of pre-existing cross-reactive antibodies targeting the S2 subunit in human individuals (n = 140) at baseline before COVID-19 vaccination. We hypothesized that this difference in pre-existing S2 antibody levels could be due to differences in priming by commensal bacteria or other microbes and resulted in differences in antibody responses after vaccination. We used the baseline S2-targeting antibody levels to stratify individuals into three groups, high (top 25% of binding, MFI > 1557), medium (middle 50% of binding, MFI 526-1557), and low (bottom 25% of binding, MFI < 526) antibody response (Fig. 6C). These grouped samples were then measured again for S2 subunit binding 3 weeks after the first immunization to the Pfizer-BioNTech's BNT162b2 vaccine. Samples with low S2 binding before vaccination at week 0 had significantly lower levels of antibodies binding the S2 subunit after the first immunization at week 3 compared to individuals with medium or high pre-existing S2 antibody levels (Fig. 6D). We did not observe any significant differences between the groups for antibody levels to the S1 or RBD subunits of the spike protein (Supplemental Fig. 3). We also did not observe any significant correlation of S2 binding levels with the antibody levels against the four seasonal coronaviruses measured in Fig. 3. This suggested that there may be preexisting antibodies elicited by reactivity with commensal gut bacterial that cross-reacted with the S2 subunit providing higher antibody response to the S2 subunit of SARS-CoV-2 without prior infection.

## Discussion

In this study, we investigated the antibody responses of individuals with recent SARS-CoV-2 infection and determined if these antibodies could cross-react with other related strains of coronavirus. We identified that natural infection with SARS-CoV-2 produced cross-reactive antibody responses that could target other known and emerging coronaviruses. Moreover, we found that vaccination with two doses of the Pfizer-BioNTech BNT162b2 mRNA COVID-19 vaccine also elicited antibodies that could react with other SARS-like and MERS betacoronaviruses.

Consistent with previous studies, we observed that the SARS-CoV-2 spike protein shares high amino acid sequence similarity to other closely related infectious coronavirus spike proteins^1,14,54,55^. These spike protein phylogenies include several emergent bat and pangolin originating coronaviruses (Bat CoV WIV1, Bat CoV RsSHC014, Bat CoV RaTG13, and Pangolin CoV identifier QIQ54048.1), that have yet to become pandemic in nature, but are being closely monitored due to their potential high-risk^61^. Due to the similarity of these aforementioned spike proteins to SARS-CoV-2 spike protein, we speculated there would be cross-reactivity with antibody responses to these other coronaviruses for individuals exposed to SARS-CoV-2, especially for SARS-CoV-1 and the bat and pangolin variants as they are all known to be classified as clade 1 betacoronaviruses^53,54^. The fact that we found cross-reactive antibodies to these related coronaviruses after infection with SARS-CoV-2 raised the hypothesis that broadly neutralizing antibodies to multiple coronaviruses could be induced. Indeed, cross-reactive antibodies have been isolated to multiple coronavirus epitopes with diverse effector functions^62^. Further characterization of the most potent neutralizing antibodies with the broadest coverage of coronaviruses will identify the optimal epitopes to target in future vaccines and therapies.

The determination that both seropositive and vaccinated individuals had high antibody binding, not only to the targeted SARS-CoV-2, but to other coronaviruses with phylogenetically similar spike protein sequences (SARS-CoV-1, MERS-CoV, Bat CoV RaTG13) could provide a template to create more broad coronavirus therapeutics or a vaccine. These novel vaccines could elicit broadly neutralizing antibodies to protect against current and future viral threats. 21 of 24 SARS-CoV-2 seropositive samples and 3 seronegative samples had antibody responses to the distantly related MERS-CoV spike protein, indicating that previous exposure to similar coronavirus epitopes could elicit cross-reactive responses to the MERS spike protein, even though it is one of the least homologous of the coronaviruses we examined. Interestingly, we found that while the whole spike protein of Human CoV OC43 had low sequence homology to SARS-CoV-2 spike protein, the S2-78 peptide region of SARS-CoV-2 had 75% identify to the same region in Human CoV OC43. This indicated that specific epitopes in even more distantly related coronaviruses could impact the antibody response. This is supported with evidence of increased antibody responses to both seasonal coronavirus Human CoV OC43 (Fig. 3A) and MERS-CoV (Fig. 2D) after vaccination with the SARS-CoV-2 spike protein. Although still detectable, we did detect a significant decrease in cross-reactive antibodies to the seasonal coronavirus NL63 spike after vaccination. It is unclear what the reason for this shift and kinetics of these changes are over the course of COVID-19 vaccination. Previous studies have demonstrated that cross-reactive immune responses with chimeric spike protein mRNA vaccines elicited protection against a multitude of related coronaviruses and SARS-CoV-2 variants by inclusion of bivalent and trivalent epitopes from multiple locations of the spike protein sequence^45,46,62,63^. Future work characterizing the pan-coronavirus cross-reactivity of these antibodies, isolation and identification of the most immunodominant epitopes through binding, structural, functional, and sequence conservation analysis would be needed to provide strong vaccine target candidacy.

We used a SARS-CoV-2 spike peptide array and sequence homology analysis to identify epitopes on the spike protein that are both immunodominant and highly conserved among related coronaviruses. Using this approach, we identified one peptide in the S2 subunit and performed a proof-of-concept study of designing a conjugate vaccine and immunizing mice. This resulted in the induction of broadly cross-reactive antibodies in the immunized mice and clearly demonstrates that peptide conjugate vaccines could induce cross-reactive coronavirus antibody responses. Although S2-78 represented the strongest candidate through binding and conservation of sequence, the antibodies produced from immunizing the mice did not result in neutralization of SAR-CoV-2 or related SARS-CoV-1 from binding to the ACE2 receptor^58–60,64–66^. Additionally, the importance of the sequence conservation can be exemplified by the lack of antibody binding to the least conserved MERS-CoV spike protein by all S2-78 peptide vaccinated mice. However, prior studies identified that antibodies targeting this epitope had neutralizing activity and correlated with disease outcomes after SARS-CoV-2 infection^58–60^. We did observe higher antibody titers to the peptide alone compared to the native spike proteins after immunization with our peptide vaccine. This suggested that the peptide vaccine could elicit a population of antibodies that did not recognize the spike protein as it is presented on the virion and would not neutralize the virus. Alternatively, serum neutralization after immunization may not have been detected because the neutralizing antibody levels were at too low at the dilution of mouse serum that was utilized. Future studies determining the specific properties of the antibodies elicited by this peptide vaccination, including other Fc-mediated functions beyond neutralization, and studies selecting other epitopes in the spike protein that may be more optimal for vaccine protection, such as those in the RBD, will be required.

There are two preliminary reports that found that antibodies elicited to SARS-CoV-2 could cross-react with proteins in the commensal gut microbiome^51,52^. One of these epitopes overlapped with the S2-78 peptide sequence we used for our immunization study. We also found high sequence similarity between S2-78 peptide and proteins found in bacteria. Moreover, we found that individuals with higher levels of pre-existing S2 antibodies before COVID-19 vaccination had significantly higher levels after immunization. Prior studies with influenza vaccines have shown that treatment with antibiotics and disruption of the microbiome may result in impairment of immunoglobulin neutralization^49^. These observations raised the hypothesis that the composition of the microbiome could influence antibody levels. Our findings identified high sequence conservation of SARS-like coronavirus spike proteins in viruses and bacteria and ascertained the possibility of the Pfizer-BioNTech BNT162b2 vaccine to boost immunity against more than just SARS-CoV-2. Future studies of components of both commensal gut bacteria and the virome should be carried out to determine how exposure to other bacteria and viruses impact the SARS-CoV-2 antibody response. A limitation of these findings is whether a neutralizing response occurs from gut bacteria exposure or if these antibodies recognize intact microbiome or if bacterial lysis must occur. Lastly, in the pursuit of generating cross-reactive antibody responses that could react with the microbiome, determinization of deleterious effects towards the microbiome or host proteins should be measured.

In summary, our data demonstrated that SARS-CoV-2 infection or vaccination elicited cross-reactive antibodies that target not only SARS-CoV-2, but related coronaviruses. Moreover, we showed that immunization with conserved peptide regions of SARS-CoV-2 induce cross-reactive antibodies in mice. Lastly, we provided evidence that suggested that the makeup of the gut microbiome could influence SARS-CoV-2 antibody levels. These data lay the groundwork for developing a pan-coronavirus vaccine that could elicit cross-reactive immunity to a broad range of coronavirus species. Further research will be needed to optimize this process in order to identify epitopes and vaccine platforms that induce protective immunity.

## Materials and methods

### Phylogenetic construction

Spike protein amino acid sequences, for the respective virus, were collected from the NCBI protein database (Supplemental Table 3). Multiple Sequence alignment (MSA) was done using MUSCLE (v3.8.1551)^67^.

RAXML (v2.0) was used for generating maximum likelihood phylogenetic tree with Blosum62 substitution model and 100 replicates^68^. Final visualization of phylogenetic tree was done using Environment for Tree Exploration (ETE) toolkit^69^.

### Human subjects

Informed consent was obtained from all subjects and/or their legal guardian(s). This study was reviewed and approved by the Children’s Mercy IRB (#00001670 and #00001317). Participants self-enrolled after they had reviewed a study information letter and were given opportunity to ask questions. All methods were carried out in accordance with relevant guidelines and regulations as reviewed and approved by the IRB. Healthcare workers from our children’s hospital were enrolled prior to the administration of the Pfizer BNT162b2 SARS-CoV-2 vaccine. Plasma from peripheral blood was collected before vaccination as a baseline (week 0), after primary immunization (week 3), and after second immunization (week 7) from individuals with no known history of infection (n = 140). Sample population consisted of mostly adult middle aged, white, females who did not identify as Hispanic or Latino (Supplementary Table 1). Pfizer-BioNTech BNT162b2 vaccine biospecimens were collected under a research study at Children’s Mercy Kansas City.

COVID-19 convalescent biospecimens were obtained through Precision for Medicine (ProMedDx, LLC, Notron, MA, USA) and were collected under a clinical study that has been reviewed by an Institutional/Independent Review Board (IRB) and/or Independent Ethics Committee (IEC) in accordance with requirements of local governing regulatory agencies including the Department of Health and Human Services (DHHS) and Food and Drug Administration (FDA) Codes of Federal Regulations, on the Protection of Human Subjects (45 CFR Part 46 and 2l CFR Part 56, respectively). 24 convalescent individuals that had PCR laboratory-confirmed SARS-CoV-2 infection were purchased from Precision for Medicine (Bethesda, MD, USA). Serum or plasma was isolated from venous whole blood collection and stored frozen in ultra-low temperature freezers until used to perform immunoassays.

### Enzyme-linked immunosorbent assays for spike antigens

ELISAs were performed using the following antigens: SARS-CoV-1 (Cat# 10683-CV, Lot# DOXT0120121, R&D Systems, Minneapolis, MN, USA), SARS-CoV-2 (Cat# 10549-CV, Lot# DODR0221011, R&D Systems), Bat CoV RaTG13 (Cat# 10660-CV, Lot# DOWW0120121, R&D Systems), MERS-CoV spike protein (Cat# 40069-V08B, Lot# LC14AP2305, Sino Biological, Wayne, PA, USA), Human CoV NL63 spike protein (Cat# 40600-V08H, Lot#LC14NO2607, Sino Biological), Human CoV 229E spike protein (Cat# 40605-V08H, Lot# LC14AP2302, Sino Biological), Human CoV HKU1 spike protein (Cat# 40021-V08H, Lot# LC14AP2707, Sino Biological), Human CoV OC43 spike protein (Cat# 40607-V08H, Lot#LC14DE1609, Sino Biological) were all diluted to 1 ug/mL in 0.1 M sodium bicarbonate and incubated on high-binding plates (3369, Corning Inc, Corning, NY, USA) overnight at 4 degrees. Serum or plasma was diluted to 1:30 in superblock buffer with sodium azide followed by subsequent 1:3 dilutions until a final dilution of 1:21,870. Secondary antibodies were purchased from Jackson ImmunoResearch (West Grove, PA, USA) : Goat anti-mouse IgG (Cat# 115-035-003, Lot# 153294) and Goat anti-human IgG (Cat# 109-036-098, Lot# 149163). Secondary antibody dilutions were done in superblock buffer without sodium azide within range of manufacturer’s recommendations at 1:50,000 dilution. SureBlue Reserve Microwell Substrate (95059-294, VWR, Radnor, PA, USA) was added and incubated in the dark for 15 min. Absorbance was measured at 450 nm immediately after 0.33 N HCl Acid Stop solution was added to the plate. Positivity threshold was determined using three times the OD450 of a negative control well without plasma.

### Manufactured ELISA

Detection and quantification of S1 IgG class antibodies was performed using the High-Sensitivity SARS-CoV-2 S1 IgG ELISA kit (41A232, Biovendor, Asheville, NC, USA) following standard protocol with serum or plasma diluted of 1:100.

### SARS-CoV-2 spike peptide array

Plasma samples were diluted 1:200 and used to probe a single SARS-CoV-2 protein and peptide microarray (CDI Labs, Mayaguez, Puerto Rico). After probing arrays with serum antibodies, the arrays were washed, labeled with an Alexa 647 anti-human IgG Fc secondary antibody and scanned using a GenePix 4000B scanner (Molecular Devices, San Jose, CA, USA). Array data was collected using the MAGPIX software (Innopsys, Chicago, IL, USA). Each protein or peptide was represented in triplicate on the microarray. There were positive control proteins (human IgG, anti-human IgG, and ACE2_Fc) and blank wells served as negative controls. The signal intensity was measured in the detection channel 635 nm (F635). The average of the F635 for each peptide was calculated and log2 transformed for graphical presentation.

Spike protein sequence from the seven different strains (Bat CoV RaTG13, Pangolin coronavirus, SARS-CoV-2, SARS-CoV-1, MERS-CoV, Bat CoV RsSHC014, BAT CoV WIV1) of coronavirus were used as reference database. Amino acid sequences for Peptide ID (S1-1 to S2-97) were then blasted against this reference database. Blastp-task blastp-short (blast-v2.2.29+) was used to find sequence homology between viral sequences and peptide of interest^70^. Only the top hit for each peptide of interest were kept for down streaming analysis. If there were two or more hits for a single peptide, within a coronavirus strain, the one with the highest length of alignment (12 base pairs) and lowest Expect value was kept. The reported E-value, percent identity and bitscore are mean scores across all seven coronavirus strains for each Peptide ID.

### S2-78 peptide synthesis

Two versions of the S2-78 peptides were synthesized (Creative Biolabs, Shirley, NY, USA). A full length 12 amino acid S2-78 peptide was synthesized for immunoassays. Additionally, a S2-78 peptide was synthesized with a conjugated CRM197 diphtheria toxin carrier protein for immunization studies to enhance the immunogenicity of the peptide vaccine.

### Mouse immunization with SARS-CoV-2 peptide conjugate vaccine

The following methods reported are in accordance with the ARRIVE guidelines (https://arriveguidelines.org) for the reporting of animal experiments. The mouse study was performed at Hooke Laboratories, Lawrence, MA, USA. All methods were reviewed and approved by the Hooke and Children’s Mercy IACUC and performed in according to the relevant international laboratory animal welfare guidance and regulations. Mice were sourced from Taconic Biosciences, Rensselaer, NY, USA.

5 BALB/c female mice between 7 and 10 weeks of age were immunized subcutaneously with 0.1 ml/mouse at 37.5 ug/mL of the FKEELDKYFKNHC-CRM197 peptide (Creative Biolabs), mixed 1:1 with Addavax (VAC-ADX-10, Invivogen, San Diego, CA, USA) on days 0, 21, and 35. Serum was collected on day 0, 28, and 42. Baseline sample (day 0) was used as the negative control for each mouse. No randomization or blinding was utilized in this study. Antibody level outcomes were performed across the timepoints during immunization and tested for statistical significance using nonparametric tests due to the exploratory sample size.

### SARS-CoV-2 viral antigen multiplexed binding assay

To measure antibody levels to SARS-CoV-2 spike subunit proteins, spike subunit 1 (S1), spike subunit 2 (S2), and receptor-binding domain (RBD), were used on a bead-based multiplex assay based on the Luminex xMAP technology. Reagent kits with secondary antibodies specific for immunoglobulin G (IgG) were used (HC19SERG1-85K, MilliporeSigma, Burlington, MA, USA) following manufacture protocol. The kit provided a set of SARS-CoV-2 antigen conjugated beads (S1, S2, RBD) along with 3 positive control beads and a negative control bead set. The positive control beads were beads coated with different concentrations of IgG. The negative control beads did not have antigen conjugated to determine nonspecific binding. The 3 antigen-conjugated beads, 3 positive control beads, and 1 negative control beads were mixed and incubated with each plasma sample at a dilution of 1:100 with assay buffer. Samples were run in duplicate. Each plate contained at least two wells with only buffer and no plasma to determine background activity. PE-anti-human IgG conjugate detection antibody was utilized to determine antibody response to each SARS-CoV-2 antigen. Using the positive control beads, inter-assay (plate-to-plate) co-efficient of variation (CV) was determined to be 5.16% for IgG. We utilized the Luminex analyzer (MAGPIX) and Luminex xPONENT acquisition software to acquire and analyze data. After acquisition net MFI was calculated by subtracting background MFI (no plasma).

After net MFI was determined for all samples, individuals before vaccination at week 0 were grouped based on their binding to S1, S2, and RBD and categorized into low (bottom 25% of binding), medium (middle 50% of binding), and high (top 25% of binding) for each antigen. These groupings were then tracked on their response at week 3 after vaccination to determine if initial binding to antigen determined future antibody response to each antigen.

### Collection of commensal gut bacterial protein from human fecal sample

A human fecal sample (D6323, Zymo Research, Irvine, CA, USA) was centrifuged at 200 g for 5 min twice to remove debris. Supernatant was collected and centrifuged at 9000 g for 10 min to collect bacterial pellet. Pellet was resuspended in PBS with 1X Halt protease and phosphatase inhibitor (78442, ThermoFisher, Waltham, MA, USA) and sonicated for 30 s with a probe sonicator 3 times. Protein concentrations were measured using the Pierce BSA protein assay kit (23225, ThermoFisher).

### Commensal gut bacteria ELISA

ELISA was performed using bacteria lysate collected from human fecal samples diluted to 1 ug/mL in 0.1 M sodium bicarbonate and incubated on high-binding plates (3369, Corning) overnight at 4 degrees. Mouse serum was diluted to 1:5 in superblock buffer with sodium azide followed by subsequent dilutions at 1:10, 1:30, 1:90, and 1:270. Secondary antibodies were purchased from Jackson ImmunoResearch: Goat anti-mouse IgG (Cat# 115-035-003, Lot# 153294). Secondary antibody dilutions were done in superblock buffer without sodium azide within range of manufacturer’s recommendations at 1:50,000 dilution. SureBlue Reserve Microwell Substrate (95059-294, VWR) was added and incubated in the dark for 15 min. Absorbance was measured at 450 nm immediately after 0.33 N HCl Acid Stop solution was added to the plate.

### Statistical analysis

The statistical analysis was performed using Graphpad Prism 9.1. For multiple comparison, the statistical significance was determined with a Wilcoxon–Mann–Whitney test with two-tailed *p* values.

## Supplementary Information

Supplementary Information.

## Data Availability

All relevant data are within the manuscript and supplementary information files. Any additional data is available from the corresponding author at reasonable request.

## References

[1] Li F (2016). Structure, function, and evolution of coronavirus spike proteins. Annu. Rev. Virol..

[2] Wu F (2020). A new coronavirus associated with human respiratory disease in China. Nature.

[3] Zhu N (2020). A novel coronavirus from patients with pneumonia in China, 2019. N. Engl. J. Med..

[4] Kirchdoerfer RN (2016). Pre-fusion structure of a human coronavirus spike protein. Nature.

[5] Walls AC (2016). Cryo-electron microscopy structure of a coronavirus spike glycoprotein trimer. Nature.

[6] Liu X (2021). Neutralizing aptamers block S/RBD-ACE2 interactions and prevent host cell infection. Angew. Chem. Weinheim Bergstr. Ger..

[7] Figueiredo-Campos P (2020). Seroprevalence of anti-SARS-CoV-2 antibodies in COVID-19 patients and healthy volunteers up to 6 months post disease onset. Eur. J. Immunol..

[8] Rodda LB (2021). Functional SARS-CoV-2-specific immune memory persists after mild COVID-19. Cell.

[9] Yuan M, Liu H, Wu NC, Wilson IA (2021). Recognition of the SARS-CoV-2 receptor binding domain by neutralizing antibodies. Biochem. Biophys. Res. Commun..

[10] Carrillo J (2021). Humoral immune responses and neutralizing antibodies against SARS-CoV-2; implications in pathogenesis and protective immunity. Biochem. Biophys. Res. Commun..

[11] Zhao J (2020). Antibody responses to SARS-CoV-2 in patients with novel coronavirus disease 2019. Clin. Infect. Dis..

[12] Long QX (2020). Antibody responses to SARS-CoV-2 in patients with COVID-19. Nat. Med..

[13] Premkumar L (2020). The receptor binding domain of the viral spike protein is an immunodominant and highly specific target of antibodies in SARS-CoV-2 patients. Sci. Immunol..

[14] Lei KC, Zhang XD (2020). Conservation analysis of SARS-CoV-2 spike suggests complicated viral adaptation history from bat to human. Evol. Med. Public Health.

[15] Jiang S, Hillyer C, Du L (2020). Neutralizing antibodies against SARS-CoV-2 and other human coronaviruses. Trends Immunol..

[16] Chia WN (2020). Serological differentiation between COVID-19 and SARS infections. Emerg. Microbes Infect..

[17] Grifoni A (2020). A sequence homology and bioinformatic approach can predict candidate targets for immune responses to SARS-CoV-2. Cell Host Microbe.

[18] Drosten C (2003). Identification of a novel coronavirus in patients with severe acute respiratory syndrome. N. Engl. J. Med..

[19] de Groot RJ (2013). Middle East respiratory syndrome coronavirus (MERS-CoV): Announcement of the Coronavirus Study Group. J. Virol..

[20] Drexler JF, Corman VM, Drosten C (2014). Ecology, evolution and classification of bat coronaviruses in the aftermath of SARS. Antiviral Res..

[21] Hilgenfeld R, Peiris M (2013). From SARS to MERS: 10 years of research on highly pathogenic human coronaviruses. Antiviral Res..

[22] Zaki AM, van Boheemen S, Bestebroer TM, Osterhaus AD, Fouchier RA (2012). Isolation of a novel coronavirus from a man with pneumonia in Saudi Arabia. N. Engl. J. Med..

[23] Zhang Y, Wen J, Li X, Li G (2021). Exploration of hosts and transmission traits for SARS-CoV-2 based on the k-mer natural vector. Infect. Genet. Evol..

[24] Zhang S (2021). Bat and pangolin coronavirus spike glycoprotein structures provide insights into SARS-CoV-2 evolution. Nat. Commun..

[25] Hamre D, Procknow JJ (1966). A new virus isolated from the human respiratory tract. Proc. Soc. Exp. Biol. Med..

[26] McIntosh K, Dees JH, Becker WB, Kapikian AZ, Chanock RM (1967). Recovery in tracheal organ cultures of novel viruses from patients with respiratory disease. Proc. Natl. Acad. Sci. U. S. A..

[27] Lau SK (2006). Coronavirus HKU1 and other coronavirus infections in Hong Kong. J. Clin. Microbiol..

[28] Woo PC (2005). Characterization and complete genome sequence of a novel coronavirus, coronavirus HKU1, from patients with pneumonia. J. Virol..

[29] van der Hoek L, Pyrc K, Berkhout B (2006). Human coronavirus NL63, a new respiratory virus. FEMS Microbiol. Rev..

[30] van der Hoek L (2004). Identification of a new human coronavirus. Nat. Med..

[31] Polack FP (2020). Safety and efficacy of the BNT162b2 mRNA Covid-19 vaccine. N. Engl. J. Med..

[32] Baden LR (2021). Efficacy and safety of the mRNA-1273 SARS-CoV-2 vaccine. N. Engl. J. Med..

[33] Sadoff J (2021). Safety and efficacy of single-dose Ad26.COV2.S vaccine against Covid-19. N. Engl. J. Med..

[34] Khoury DS (2021). Neutralizing antibody levels are highly predictive of immune protection from symptomatic SARS-CoV-2 infection. Nat. Med..

[35] Bradley T (2021). Antibody responses after a single dose of SARS-CoV-2 mRNA vaccine. N. Engl. J. Med..

[36] Fraley E (2021). Humoral immune responses during SARS-CoV-2 mRNA vaccine administration in seropositive and seronegative individuals. BMC Med..

[37] Corbett KS (2020). Evaluation of the mRNA-1273 vaccine against SARS-CoV-2 in nonhuman primates. N. Engl. J. Med..

[38] Mercado NB (2020). Single-shot Ad26 vaccine protects against SARS-CoV-2 in rhesus macaques. Nature.

[39] Vogel AB (2021). BNT162b vaccines protect rhesus macaques from SARS-CoV-2. Nature.

[40] Fraley E (2021). Cross-reactive antibody immunity against SARS-CoV-2 in children and adults. Cell. Mol. Immunol..

[41] Song, G. *et al.* Cross-reactive serum and memory B cell responses to spike protein in SARS-CoV-2 and endemic coronavirus infection. *bioRxiv*. 10.1101/2020.09.22.308965 (2020). 10.1038/s41467-021-23074-3 PMC8134462 34011939

[42] Ng KW (2020). Preexisting and de novo humoral immunity to SARS-CoV-2 in humans. Science.

[43] Muthumani K (2021). Preexisting vs. de novo antibodies against SARS-CoV-2 in individuals without or with virus infection: Impact on antibody therapy, vaccine research and serological testing. Transl. Med. Commun..

[44] Majdoubi A (2021). A majority of uninfected adults show preexisting antibody reactivity against SARS-CoV-2. JCI Insight.

[45] Saunders KO (2021). Neutralizing antibody vaccine for pandemic and pre-emergent coronaviruses. Nature.

[46] Martinez DR (2021). Chimeric spike mRNA vaccines protect against Sarbecovirus challenge in mice. Science.

[47] Eickhoff CS (2019). Highly conserved influenza T cell epitopes induce broadly protective immunity. Vaccine.

[48] Singh S (2016). Heterologous Immunity between Adenoviruses and Hepatitis C Virus: A new paradigm in HCV immunity and vaccines. PLoS ONE.

[49] Hagan T (2019). Antibiotics-driven gut microbiome perturbation alters immunity to vaccines in humans. Cell.

[50] Williams WB (2015). HIV-1 VACCINES. Diversion of HIV-1 vaccine-induced immunity by gp41-microbiota cross-reactive antibodies. Science.

[51] Jia, L. W. S., Wu, J., Tian, X., Zhang, Y., Wang, X., Wang, J., Yan, D., Wang, W., Zhu, Z., Qiu, C., Zhang, W., Xu, Y. & Wan, Y. Pre-existing antibodies targeting a linear epitope on SARS-CoV-2 S2 cross-reacted with commensal gut bacteria and shaped vaccine induced immunity. *medRxiv*. 10.1101/2021.07.13.21260404 (2021).

[52] Ninnemann, J. B. L., Bondareva, M., Witkowski, M., Angermair, S., Kreye, J., Durek, P., Reincke, S. M., Sánchez-Sendin, E., Yilmaz, S., Sempert, T., Heinz, G. A., Tizian, C., Raftery, M., Schönrich, G., Matyushkina, D., Smirnov, I. V., Govorun, V. M., Schrezenmeier, E., Dörner, T., Zocche, S., Viviano, E., Sehmsdorf, K. J., Chang, H., Enghard, P., Treskatsch, S., Radbruch, A., Diefenbach, A., Prüss, H., Mashreghi, M. & Kruglov A. A. Induction of cross-reactive antibody responses against the RBD domain of the spike protein of SARS-CoV-2 by commensal microbiota. *bioRxiv*. 10.1101/2021.08.08.455272 (2021).

[53] Lu R (2020). Genomic characterisation and epidemiology of 2019 novel coronavirus: Implications for virus origins and receptor binding. Lancet.

[54] Petrosillo N, Viceconte G, Ergonul O, Ippolito G, Petersen E (2020). COVID-19, SARS and MERS: Are they closely related?. Clin. Microbiol. Infect..

[55] Cui J, Li F, Shi ZL (2019). Origin and evolution of pathogenic coronaviruses. Nat. Rev. Microbiol..

[56] Ye ZW (2020). Zoonotic origins of human coronaviruses. Int J Biol Sci.

[57] Paules CI, Marston HD, Fauci AS (2020). Coronavirus infections—More than just the common cold. JAMA.

[58] Li Y (2020). Linear epitopes of SARS-CoV-2 spike protein elicit neutralizing antibodies in COVID-19 patients. Cell. Mol. Immunol..

[59] Li Y (2021). Systematic evaluation of IgG responses to SARS-CoV-2 spike protein-derived peptides for monitoring COVID-19 patients. Cell. Mol. Immunol..

[60] Poh CM (2020). Two linear epitopes on the SARS-CoV-2 spike protein that elicit neutralising antibodies in COVID-19 patients. Nat. Commun..

[61] Menachery VD (2015). A SARS-like cluster of circulating bat coronaviruses shows potential for human emergence. Nat. Med..

[62] Shiakolas AR (2021). Cross-reactive coronavirus antibodies with diverse epitope specificities and Fc effector functions. Cell. Rep. Med..

[63] Shen X (2021). SARS-CoV-2 variant B.1.1.7 is susceptible to neutralizing antibodies elicited by ancestral spike vaccines. Cell Host Microbe.

[64] Ma ML (2021). Systematic profiling of SARS-CoV-2-specific IgG responses elicited by an inactivated virus vaccine identifies peptides and proteins for predicting vaccination efficacy. Cell. Discov..

[65] Barrett CT (2021). Effect of clinical isolate or cleavage site mutations in the SARS-CoV-2 spike protein on protein stability, cleavage, and cell–cell fusion. J. Biol. Chem..

[66] Xia S (2020). Inhibition of SARS-CoV-2 (previously 2019-nCoV) infection by a highly potent pan-coronavirus fusion inhibitor targeting its spike protein that harbors a high capacity to mediate membrane fusion. Cell Res..

[67] Edgar RC (2004). MUSCLE: A multiple sequence alignment method with reduced time and space complexity. BMC Bioinform..

[68] Edler DKJ, Antonelli A, Silvestro D (2020). raxmlGUI 2.0: A graphical interface and toolkit for phylogenetic analyses using RAxML. Methods Ecol. Evol..

[69] Huerta-Cepas J, Serra F, Bork P (2016). ETE 3: Reconstruction, analysis, and visualization of phylogenomic data. Mol. Biol. Evol..

[70] Altschul SF (1997). Gapped BLAST and PSI-BLAST: A new generation of protein database search programs. Nucl. Acids Res..

