# Supplementary material for: Cross-reactive antibodies elicited to conserved epitopes on SARS-CoV-2 spike protein after infection and vaccination
Source: Sci Rep. 2022 Apr 20;12:6496. doi: 10.1038/s41598-022-10230-y (PMC9019795; doi:10.1038/s41598-022-10230-y)
Supplement: Supplementary file 1 — Supplementary Information. [file 41598_2022_10230_MOESM1_ESM.pdf]

Cross-reactive antibodies elicited to conserved epitopes on SARS-CoV-2 spike protein after infection and vaccination. Supplemental Material.

Eric S. Geanes, Cas LeMaster, Elizabeth R. Fraley, Santosh Khanal, Rebecca McLennan, Elin Grundberg, Rangaraj Selvarangan, Todd Bradley

Supplemental Figure 1: Sequence homology of SARS-CoV-2 sequences to other coronaviruses.
Supplemental Figure 2: SARS-CoV pseudovirus neutralization.
Supplemental Figure 3: S1 and RBD Binding levels.

Supplemental Table 1: Seropositive participant demographics
Supplemental Table 2: Vaccinated participant demographics
Supplemental Table 3: Spike protein Phylogeny database information

A

Virus	Amino acid identity to SARS-CoV-2 (%)
Bat CoV RaTG13	97.408
Pangloin CoV QIQ54048.1	92.145
Bat CoV RsSHC014	77.188
Bat CoV WIV1	76.953
SARS-CoV-1	76.038
MERS-CoV	30.808
Human_CoV_OC43	29.536
Human_CoV_HKU1	28.421
Human_Cov_NL63	25.447
Human_CoV_229E	25.289

B

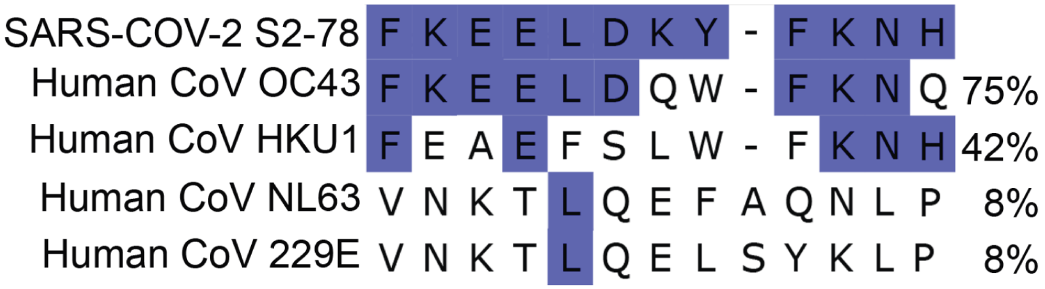

Supplemental Figure 1. Sequence homology of SARS-CoV-2 sequences to other coronaviruses. Sequence alignments of **(A)** full spike protein **(B)** S2-78 peptide sequence of SARS-CoV-2 to emergent and seasonal coronaviruses. Amino acid identity displayed in percentage. Blue shading indicated identity with SARS-CoV-2.

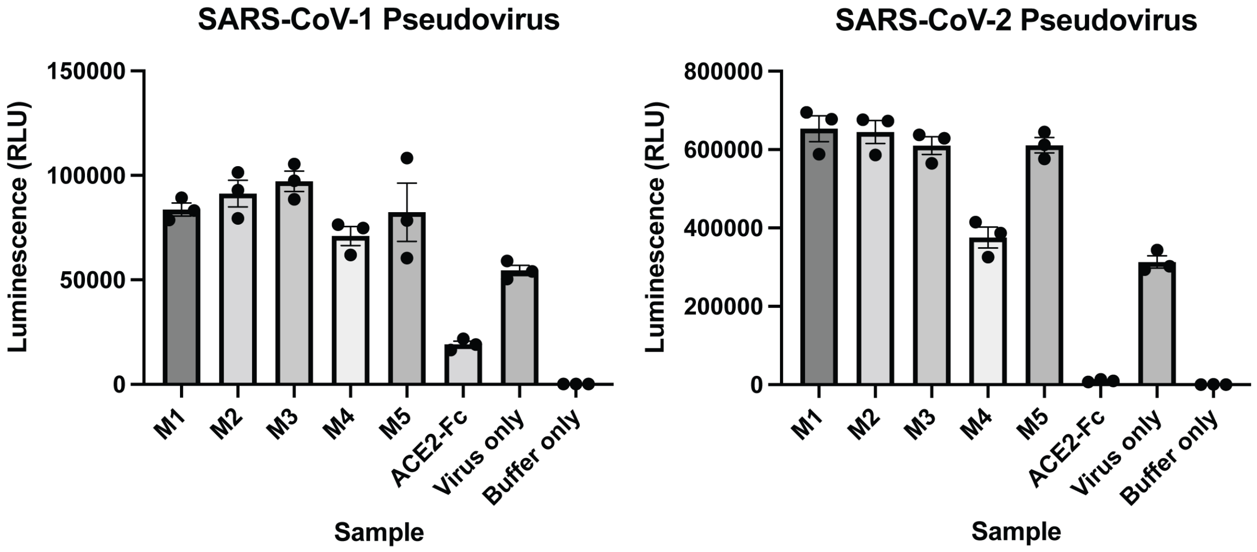

Supplemental Figure 2. SARS-CoV pseudovirus neutralization. Immunized mouse serum from day 42 was tested for ability to neutralize SARS-CoV-1 or SARS-CoV-2 spike pseudoviruses that had luciferase reporter gene from infecting human ACE2 overexpressing HEK-293 cells (Genscript). All assays were performed in technical triplicate. Five mice serum were tested (M1-M5) at 1:40 serum dilution, ACE2-Fc positive control and wells with pseudovirus or buffer only as negative controls. Reduction in RLU indicates neutralization.

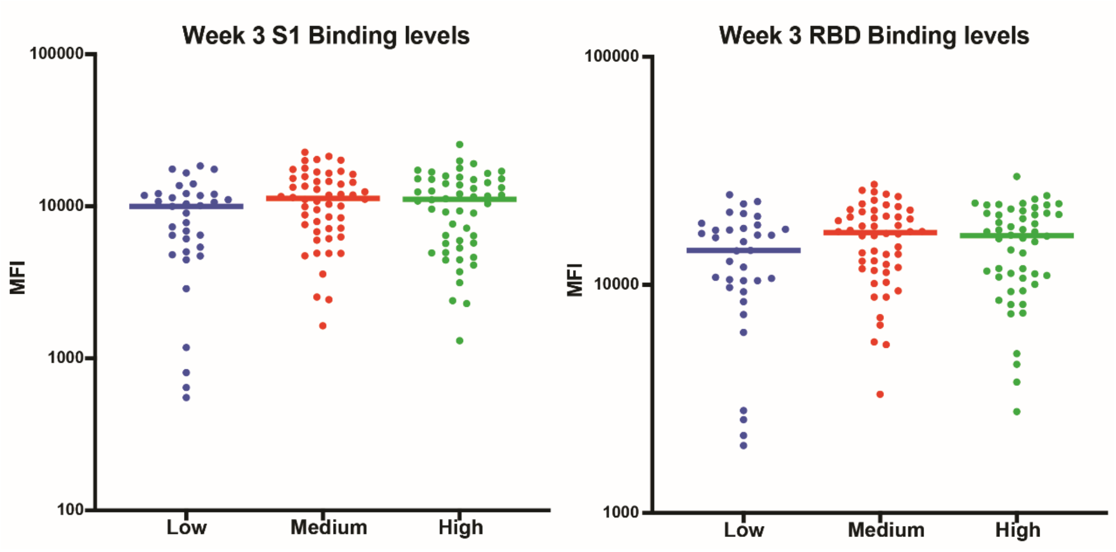

Supplemental Figure 3. S1 and RBD Binding levels. Multiplex bead-based antibody binding assay that measured the IgG antibody response to SARS-CoV-2 spike subunit 1 (S1) and the receptor-binding domain (RBD) in individuals at week 3 after receiving the first COVID-19 vaccine immunization. Individual values of Median Fluorescent Intensity (MFI) are calculated; background subtraction has been used to remove nonspecific signal. Bar indicates group median. Individuals were grouped using their baseline (week 0) binding to the S2 subunit. The top 75% MFI were grouped as high (green) and individuals with the lowest 25% MFI were grouped as low (blue) and individuals with 25%-75% MFI were grouped as medium (red). P values were determined with a Wilcoxon-Mann-Whitney test.

Supplemental Table 1: Seropositive participant demographics

	**Seropositive** (COVID19+; N=24)
**Age**	Median: 44 years old<br>Range: 28-67 years old
**Gender**	Male: 10<br>Female: 14
**Race**	White: 4<br>Black or African Amercian: 2<br>Hispanic: 16<br>American Indian or Alaska Native: 0<br>Asian: 1<br>Native Hawaiian or Other Pacific Islander: 0<br>Multiracial: 0<br>Unknown: 1
**Ethnicity**	Not Recorded
**Infection**	Median: 35 Days prior to collection<br>Range: 12-42 Days prior to collection

Supplemental Table 2: Vaccinated participant demographics

	**Seronegative** (COVID19-; N=152)
**Age**	Median: 46 years old<br>Range: 22-75 years old
**Gender**	Male: 46<br>Female: 106
**Race**	White: 133<br>Black or African Amercian: 3<br>American Indian or Alaska Native: 0<br>Asian: 7<br>Native Hawaiian or Other Pacific Islander: 0<br>Multiracial: 5<br>Unknown: 4
**Ethnicity**	Hispanic or Latino: 10<br>Not Hispanic or Latino: 129<br>Unknown: 13

Supplemental Table 3: Spike protein Phylogeny database information

Accession ID	Organism	Definition	Reference	NCBI Database
QEG03814.1	Human CoV OC43	spike protein [Human coronavirus OC43]	GenBank	Protein
BBA20986.1	Human CoV HKU1	spike protein [Human coronavirus HKU1]	GenBank	Protein
AWH62679.1	Human CoV 229E	spike protein [Human coronavirus 229E]	GenBank	Protein
APF29063.1	Human Cov NL63	spike protein [Human coronavirus NL63]	GenBank	Protein
QHR63300.2	Bat coronavirus RaTG13 or bat_CoV_TG13	spike glycoprotein [Bat coronavirus RaTG13]	GenBank	Protein
QIQ54048.1	Pangolin_coronavirus	spike protein [Pangolin coronavirus]	GenBank	Protein
YP_009724390.1	SARSCoV2	surface glycoprotein [Severe acute respiratory syndrome coronavirus 2]	NCBI Reference Sequence	Protein
AAP13441.1	SARSCoV1	S protein [SARS coronavirus Urbani]	GenBank	Protein
YP_009047204.1	MERSCoV	spike protein [Middle East respiratory syndrome-related coronavirus]	NCBI Reference Sequence	Protein
AGZ48806.1	Bat CoV RsSHC014	spike protein [Bat SARS-like coronavirus RsSHC014]	GenBank	Protein
AGZ48831.1	Bat CoV WIV1	spike protein [Bat SARS-like coronavirus WIV1]	GenBank	Protein
